# Movable Kidney

**Published:** 1896-01-04

**Authors:** 


### PROGRESS IN SURGERY.

### MOVABLE KIDNEY.

Considerable light has been thrown on this condition during the last few years.

**Causation.**—Kendal Franks[1] shows that the true shape of the kidney during life is a rude double cone. This was proved by Professor Cunningham, who injected unopened bodies gradually with hardening fluid; the shape of the kidneys and the indentations on their surface produced by pressure of adjacent organs are thus preserved. These pressures on the double cone are roughly from above and below with a resultant forcing the gland backwards. When these forces are in equilibrium the normal position is maintained; if, therefore, the upward pressure of the bowels is unusual, or increased pressure produced from above, the equilibrium may be disturbed, never to be re-established. This has been roughly known for some years, but K. Franks' paper enforces the purely mechanical view of the problem.

The changes which lessen the upward pressure are variations in the size of the abdominal contents, as flatulence, child-bearing, ovariotomy, and yielding of the pelvic floor. Increased pressure in the downward direction is afforded by tight lacing, enlargement of the liver, and enlargement of the gall bladder.[2] The frequent coexistence of jaundice with movable kidney is marked.[3]

H. Morris points out that (1) movable kidney and gall stone are both far commoner in women. (2) The right kidney is far more commonly affected. Of 173 cases (according to Landaur) the right kidney was affected in 152, the left in 12, and both in 9. (3) Gallstones and movable kidney are often co-existent. Morris's cases show no influence of child-bearing, though he does not exclude the other factors of varying abdominal pressure. Moreover, he fails to note that a liver which eventually produces gallstones will probably be intermittently engorged, and will therefore exert increased downward pressure by general increase of size. He ascribes the three points stated to wearing stays.

The relation of hydronephrosis to movable kidney is a debated question still. Movable kidneys are frequently found the seat of hydronephrosis. Jordan Lloyd,[4] and others, have assumed that the hydronephrosis, by its increase of weight, produces the mobility. On the other hand, Clement Lucas,[5] following Landaur, teaches that the mobility of the kidneys produces the hydronephrosis. For a very slight rotatory displacement of the kidney will so twist the vessels that the thin-walled vein will prevent egress of blood, while the stouter artery permits a larger ingress, hence arises an intermittent engorgement which will in time produce hydronephrosis. On the other hand, the engorgement produces greater downward displacement, and so the vicious cycle goes on.

Another cause of hydronephrosis in such cases is kinking of the lax and elongated ureter.

All these factors of causations apply to kidneys which are normal in peritoneal relation, and to those which have (as a few are found to have) a mesonephron or incomplete investiture of peritoneum. The former are classed as "movable" kidney, the latter (having a more limited range of movement) as "floating" kidneys. A mesonephron may, however, be long enough to permit of movement to the wrong side of the vertebral column.[6]

**Gastric Crises.**—The attacks of sickness which occur are of importance; they may be so severe as to resemble those of locomotor ataxia.[7] In these cases dilation of the stomach is often present. Renzi[8] is doubtful if this is due to mere coincidence, to reflex neurotic disturbance, or to direct pressure. Franks[9] has no doubt that it is caused by the connective tissue attachment of the kidney to the descending portion of the duodenum; both viscera are retroperitoneal, and the kidney in its descent drags down the duodenum and kinks it, causing partial obstruction.

**Nephrorraphy.**—The operation of attaching the movable kidney was first practised by Hahn in 1883; he sutured the organ to the lumbar muscles and skin. It is usual now to attach it to the tips of the two last ribs. It is important to be sure that the ribs project beyond the lumbo-sacral muscles, otherwise the pleura may be wounded.[10]

Few surgeons now pass the sutures through the perinephric fat only, as such an attachment is too yielding; many have even sutured boldly through the kidney substance, a procedure which may be found in time to produce calculus.[11] The most reasonable plan is to suture through the fibrous capsule, stripping an area of the capsule off between the ligatures to gain better adhesion.

**Results of Operation.**—Out of 374 cases collected by Albarran there were seven deaths, only four attributed to the operation. In 88 per cent. the pain was lessened; 36 per cent. gained no relief.[12]

Any case which presents undoubted enlargement of the kidney during attacks of pain (and these attacks cannot be prevented by the pressure of a belt) should be subjected to operation. Even when hydronephrosis is far advanced, the artificial fixation may prevent its progress, and save the remainder of the gland in functional activity.

If the organ is largely disintegrated Bruce Clarke[13] advises its removal, always ascertaining that the second kidney is sound. If so, the fact that one kidney is much diseased is a guarantee that the other is able to carry on the work of both.

[1] Brit. Med. Journ., 1895, II., 895. [2] H. Morris, Brit. Med. Journ., 1895, II., 239. [3] Landaur, Wandermire der Frauen, New. Syd. Soc. Trans.; Morris loc. cit.; Hale White, Brit. Med. Journ., 1892, I., 223; Rendu, Prog. Méd., Oct. 29, 1895; Bland Sutton, Brit. Med. Journ., 1895, II., 899. [4] Practitioner, 1887, xxxix., p. 180. [5] Brit. Med. Journ., 1891, II., 1,343. [6] Oliver, Brit. Med. Journ., 1885, I., 483. [7] Prog. Méd., loc. cit. [8] Renzi, Rif. Med., Dec. 22, 1894. [9] Loc. cit. [10] Albarran, Ann. des Mal. des Org. Gen. Ur., Aug., 1895. [11] T. Smith, Trans. Medico-Chirurg. Soc., April 11, 1893. [12] See also Bruce Clarke, Brit. Med. Journ., 1895, I., 575.

